# Metatranscriptome analysis of symptomatic bitter apple plants revealed mixed viral infections with a putative novel polerovirus

**DOI:** 10.1186/s12864-024-10057-z

**Published:** 2024-02-15

**Authors:** Shahrbanou Ghorani, Hossein Massumi, Samin H. Farhangi, Mehdi Mansouri, Jahangir Heydarnejad, Akbar Hosseinipour

**Affiliations:** 1 Department of Plant Protection, College of Agriculture, Shahid Bahonar University of Kerman, Kerman, 7616914111 Iran; 2 Research and Technology Institute of Plant Production (RTIPP), Shahid Bahonar University of Kerman, Kerman, Iran; 3 Animal Breeding and Genomics, Wageningen University & Research, Wageningen, The Netherlands; 4 Department of Agricultural Biotechnology, College of Agriculture, Shahid Bahonar University of Kerman, Kerman, Iran

**Keywords:** *Citrullus colocynthis*, Next-generation sequencing, Recombination, Bitter apple aphid-borne yellows virus

## Abstract

**Background:**

Next-generation Sequencing (NGS) combined with bioinformatic analyses constitutes a powerful approach for identifying and characterizing previously unknown viral genomes. In this study, leaf samples from bitter apple plants (*Citrullus colocynthis* (L.) Schrad) exhibiting symptoms such as dwarfing, leaf crinkling, and chlorosis were collected from the southern part of Kerman province, Iran.

**Results:**

Putative infecting viruses were identified through *de novo* assembly of sequencing reads using various tools, followed by BLAST analysis. Complete genomes for Squash vein yellowing virus (SqVYV), Citrus-associated rhabdovirus (CiaRV), and a novel polerovirus-related strain termed Bitter apple aphid-borne yellows virus (BaABYV) were assembled and characterized. Additionally, a partial genome for Watermelon mosaic virus (WMV) was assembled. The genomic organization of the BaABYV was determined to be 5’-ORF0-ORF1-ORF1,2-ORF3a-ORF3-ORF3,5-ORF4-3’. Amino acid sequence identities for inferred proteins (P0 and P1, P1,2) with known poleroviruses were found to be the 90% species delineation limit, implying that BaABYV should be considered a new member of the genus *Polerovirus.* Recombination events were observed in the BaABYV and WMV strains; such events were not found in the CiaRV strain.

**Conclusions:**

Molecular evidence from this study suggests that *C. colocynthis* is a reservoir host of several plant viruses. Among them, BaABYV is proposed as a new member of the genus *Polerovirus*. Furthermore, the CiaRV strain has been reported for the first time from Iran.

**Supplementary Information:**

The online version contains supplementary material available at 10.1186/s12864-024-10057-z.

## Background

In agricultural fields, weeds contribute to increase competition for essential resources like water and nutrients. Furthermore, they can serve as reservoirs for viruses that pose infection risks to neighboring crops [1]. One such plant is *C. colocynthis*, commonly referred to as bitter apple or colocynth, which belongs to the *Cucurbitaceae* family and is adapted to arid environments. The bitter apple fruit is valued for its medicinal benefits [2] and is extensively cultivated in southern Iran [3]. Previous studies have identified several viruses in *C. colocynthis*, including *Cucurbit aphid-borne yellows virus* (CABYV), *Squash mosaic virus* (SqMV), *Papaya ring spot virus-*type W (PRSV-W), *Cucumber mosaic virus* (CMV), *Zucchini yellow mosaic virus* (ZYMV), and *Watermelon mosaic virus* (WMV). These findings suggest that wild species like *C. colocynthis* can act as a viral reservoir affecting agricultural crops [4–8].

Research on viruses in wild plants offers multiple advantages, such as enhancing our understanding of viral diversity and host-virus interactions, conserving biodiversity, and providing insights into viral evolution [9]. Next-generation Sequencing (NGS) has revolutionized this field by enabling comprehensive sequencing of all viral genomes in a plant tissue sample [10]. This high-throughput technology sequences the entire viral genome after isolating its RNA or DNA from the plant tissue, facilitating rapid identification and characterization of known and unknown viruses [11–13]. In particular, metagenomics is a valuable NGS technique for detecting unidentified plant viruses, especially in asymptomatic hosts where traditional methods may not easily detect them. Metagenomics sequences all genetic material in a sample, providing a comprehensive snapshot of the viral community present [14].

Despite the bitter apple is native to west Asia including Iran, there has been notable gap in research identifying the viruses associated with these plants. A study by Sharifi et al. [8] made significant contributions in this area by detecting the WMV in bitter apple plants in the southern Kerman province, Jiroft region. However, this research did not explore the full spectrum of viruses potentially affecting these plants. In an effort to extend the findings of earlier research, our study aims to deepen the understanding of the virome associated with bitter apple plants. Using NGS technology, we focused on a group of bitter apple plants from the Jiroft region, all exhibiting virus-like symptoms. Our study led to discovery of a mixed viral infection in bitter apple plants, including the identification of a new type of a polerovirus.

## Materials and methods

### Sample collection

**Fig. 1:**
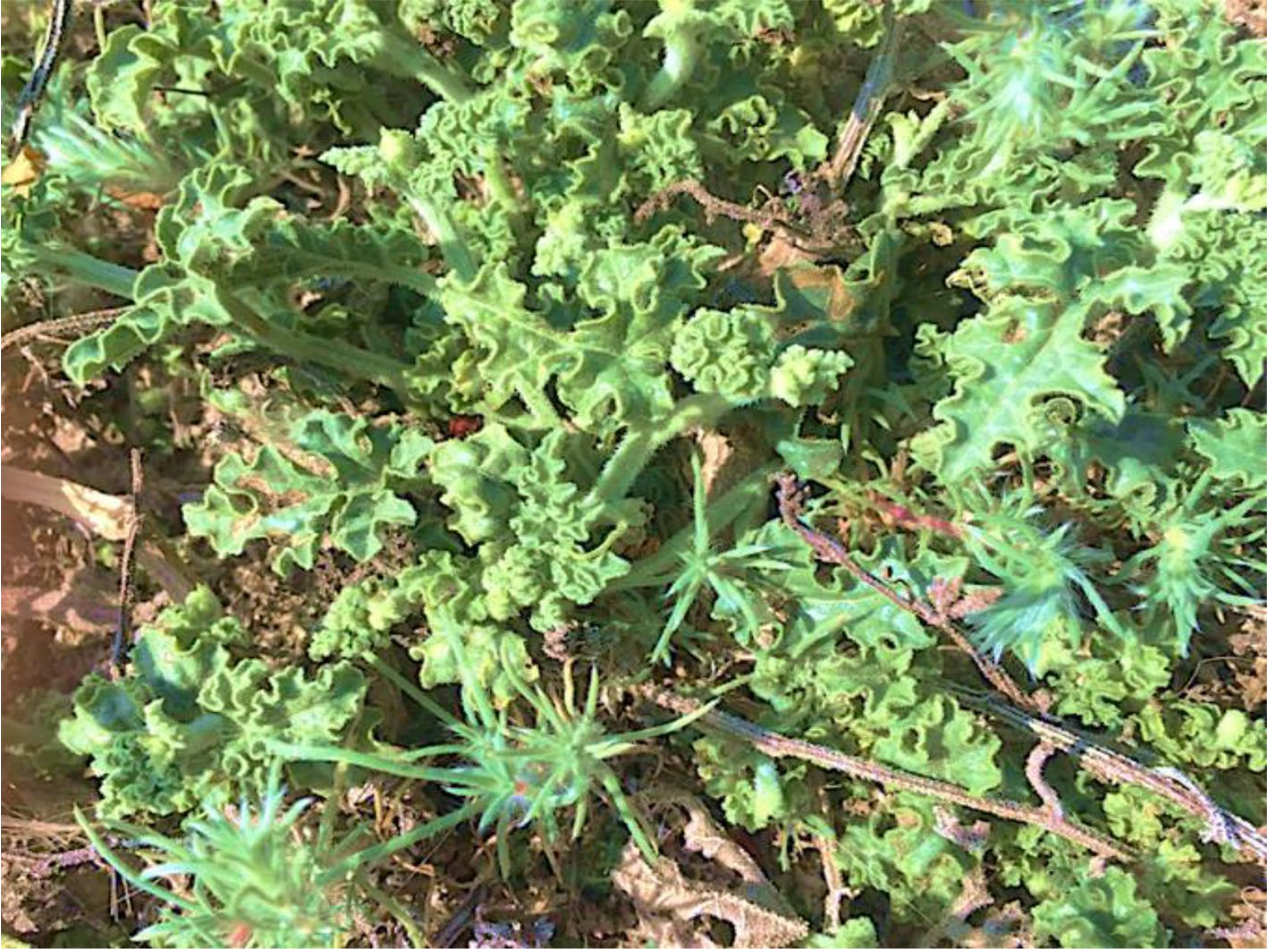
A bitter apple plant exhibiting suspected viral symptoms, including dwarfing, leaf crinkling, and mild chlorosis, in its natural habitat (Jiroft region, Kerman province, Iran)

To investigate potential viruses infecting *C. colocynthis*, we collected samples with symptoms of viral diseases, including dwarfing, leaf crinkling, and mild chlorosis (Fig. 1) from a desert area in the Jiroft region (28°26’33.4"N 57°54’17.0” E) in July 2021. The specificity of our selection criteria led us to identify a limited number of samples (*n* = 7) that clearly exhibited these viral symptoms. These samples were immediately placed in plastic bags, flash-frozen in liquid nitrogen, and transferred to the Shahid Bahonar University laboratory. The plant materials utilized in our study were identified and verified in the herbarium (MIR-4307) by Dr. S.M. Mirtadzaddini from the Department of Biology, Faculty of Science at Shahid Bahonar University of Kerman.

They were stored at -80 °C pending further analysis.

### Total RNA extraction and sequencing

A pooled sample from leaves with relatively severe symptoms was ground using a mortar and pestle. Total RNA was extracted using the TOP Plant and Fungi RNA Purification Kit (mini-prep; Cat. No; TGK2004), following the manufacturer’s instructions (Topazgen, Iran). The integrity of the RNA was confirmed by the presence of distinct bands corresponding to 28 S ribosomal RNA (rRNA) (~ 4.8 kb), 18 S rRNA (~ 2.0 kb), and 5.8 S rRNA (~ 154 nt) on an agarose gel. High-quality RNA samples were purified using kit and concentrated prior to sequencing. Novogen (Beijing, China) constructed a paired-end sequencing library using the Illumina HiSeq 6000 sequencing platform.

### Sequence analysis

The raw RNA-seq data were evaluated by FastQC v.0.11.9 [15]. Raw reads were processed using Cutadapt (Version 2.0.4) [16] to retain only those with a minimum length of 50 bases and a quality score exceeding 30; this step trimmed low-quality reads and adapter sequences. The sequencing data were then assembled using Trinity (v2.4.0) [17], SPAdes (v3.13.0) [18], and CLC Genomics Workbench (22.0.2) (CLC Bio, Aarhus, Denmark), with their default parameters. The assembled contigs were subjected to BLAST analysis against public nucleotide datasets and the Reference Viral DataBase (RVDB) (V 25.0) to identify potential viruses. The purified reads were mapped against the reference genome of the most closely related virus using NextGenMap 0.5.0 [19] to validate the results of de novo assembly approaches. Samtools flagstat (Version 2.0.4) [20] provided descriptive statistics for the BAM files and read counts for each viral reference genome.

### Phylogenetic analysis

We compiled full-genome sequences of identified viruses and aligned them independently using MAFFT Version 7.0 [21]. The nearest non-target virus was selected as an outgroup based on BLASTN results. Potential recombinant isolates have been detected using seven recombination detection methods implemented in the RDP4 package (RDP, GENECONV, BootScan, MaxChi, Chimaera, SiScan, and 3Seq) [22]. Recombinant sequences were considered valid if detected by at least four of the methods and with a *P*-value less than 10^− 6^ under the default setting for linear sequences. These identified recombinants were excluded from the multiple sequence alignments (MSAs). Subsequently, we created a maximum likelihood phylogenetic tree (MLtree) using the IQtree program [23]. The IQ-tree automatically selected the best model using the ModelFinder program implemented in the IQ-tree tool. The reconstructed consensus ML tree was visualized using the Figtree v.1.4.3 (http://tree.bio.ed.ac.uk/software/figtree). Nucleotide pairwise distances were computed using the default option in MEGAX [24].

### RT-PCR and sanger sequencing

Total RNA was extracted using a Spectrum Plant Total RNA Kit (Sigma Aldrich, USA), from the pooled sample, previously prepared for NGS analysis, which included seven bitter apple plants. For the reverse transcription (RT) reaction, a mixture was prepared consisting of 3 µL of reverse primer (20 pmol) (Table 1), 5 µL of RNA sample, and 8.5 µL of sterile deionized distilled water. The mixture was incubated at 95 °C for 1 min and cooled on ice for 3 min to denature the RNA. Subsequently, 4 µL of 5× M-MLV RT buffer, 2 µL of dNTPs mix (10 mM), 1 µL M-MLV reverse transcriptase (200 U µL), and 1 µL RNase inhibitor (20 U/µL) (Sinaclon, Iran) were added. The reaction mixture was then incubated at 42 °C for 60 min and terminated by heating at 65 °C for 10 min. For the RT-PCR, 2.5 µL of cDNA was mixed with 7.5 µL DEPC-treated water, 1.25 µL of 5× GoTag polymerase buffer, 2.5 µL of 10× MgCl2, 0.5 µL of each forward and reverse primer (20 pmol), 0.75 µL of dNTP mix (10 mM) and 0.125 µL of GoTag polymerase (2.5 U/µL) (Sinaclon, Iran). The RT-PCR program included an initial denaturation at 94 °C for 3 min, followed by 35 cycles of 30 s at 94 °C for denaturation, 30 s of annealing at temperatures specified in Tables 1 and 30s of extension at 72 °C, and concluded with a final extension step at 72 °C for 10 min.

RT-PCR products were analyzed by electrophoresis on a 1% agarose gel and visualized with ethidium bromide staining. The products were then purified using an Agarose Gel DNA Extraction Kit (Sangon, Shanghai, China) and sequenced directly with the Applied Biosystems 3500 Genetic Analyzer (Foster City, CA, USA) using the RT-PCR primers in the forward direction.

**Table 1:** PCR primers designed to detect and discriminate viruses in this study

Primer name	Sequence (5´ to 3´)	Position	PCR product size (bp)	Annealing temperature (^o^C)
BaABYV -F	TGA TCG CGA ACT ACA TGT CC	3236–3255	939	56
BaABYV -R	GGA ACT GCC GTC TAC CTA TTT	4174 − 4154		
SqVYV-F	GGA AGC ACT CAT GCC TGA TATT	8517–8538	1158	55
SqVYV-R	GGT GCT GAA CAG TAC CTC A	9674 − 9656		
CiaRV-F	TCT CCG GTA TTT GAG AAG CAC	10,973–10,993	641	52
CiaRV-R	CCG AGC AAC CCT TAT CTG TTT′	11,613 − 11,593		
WMV F	GAA TCA GTG TCT CTG CAA TCA GG	2703–2725	822	55
WMV R	ATT CAC GTC CCT TGC AGT GTG	3524 − 3504		

BaABYV (Bitter apple aphid-borne yellows virus), SqVYV (Squash vein yellowing virus), CiaRV (Citrus-associated rhabdovirus), WMV (Watermelon mosaic virus)

## Results

### Data processing and assembly

After trimming the raw reads using the Cutadapt program, 98.90% of the reads were retained for further analysis. These trimmed reads were then assembled into contigs using three different *de novo* assembly methods: (i) Trinity, (ii) SPAdes, and (iii) CLC Genomic workbench. The assembly metrics, such as contig count, maximum length, total length, minimum length, average length and N50 values, for each method are compared and summarized in Appendix Table A1. Trinity generated the highest number of contigs (77,188), while SPAdes achieved the highest N50 value (1,834), indicating the assembly of contigs with superior quality. Despite SPAdes’s higher N50, the analysis proceeded with contigs assembled by Trinity. This decision was motivated by the more significant number of contigs from Trinity, which could offer additional data richness and uncover more insights into the virome. Moreover, it could be essential for identifying low-abundance viruses or novel variants that might be missed when focusing only on high-quality contigs.

### Virus identification

All *de novo* assembled contigs from Trinity exceeding 3,500 bp were subjected to BLASTN for virus identification, searching against available nucleotide datasets and DataBase RVDB based on percentage of identity. Four contigs corresponding viruses within genera *Potyvirus*, *Polerovirus*, *Rhabdovirus*, and *Ipomovirus* were identified, with the details summarized in Table 2.

**Table 2:** The parameter values of BLASTN-aligned contigs and their two closest virus sequences

Contig name	Length of contig (nt)	Query coverage (%)	Identity (%)	E-value	Virus	Accession number
C-1	5,816	96	90.13	0.0	*Pepo aphid-borne yellows virus*	KU315178
		98	88.81	0.0	*Pumpkin polerovirus*	NC_055513
C-2	9,912	99	98.42	0.0	*Squash vein yellowing virus*	ON229619
		99	98.42	0.0	*Squash vein yellowing virus*	KT721735
C-3	3,757	100%	96.46%	0.0	*Watermelon mosaic virus*	EU660580
		100%	95.55%	0.0	*Watermelon mosaic virus*	KC292915
C-4	13,443	71	80.82%	0.0	*Citrus-associated rhabdovirus isolate C1*	MT302545
		77%	80.77%	0.0	*Citrus-associated rhabdovirus isolate C2*	MT302546

### Characterization and phylogenetic analysis of the BaABYV-IR-1 strain

Computational analyses revealed that contig C-1 from the BaABYV-IR-1 strain has a nucleotide length of 5,816. It exhibited over 90% identity with the *Pepo aphid-borne yellows virus* (PABYV), which is classified within the genus *Polerovirus* (Table 2). To validate these findings, the trimmed reads from the BaABYV-IR-1 strain were mapped to the reference genomes of PABYV (NC_030225) and pumpkin polerovirus (PuPV) (NC_055513). The mapping confirmed that 0.01% of the trimmed reads, ranging from 3,744 to 3,790 nucleotides, were re-mapped onto the reference genomes. The bioinformatic analysis highlighted the typical ORF structure of the BaABYV-IR-1 strain (the contig C-1), revealing 7 ORFs characterized in Table 3.

**Table 3:** ORF organization of contig C-1 from BaABYV-IR-1, including putative proteins and closely related strains

Predicted ORF/region	Gene products	Contigs	Length nt (aa)	Positions (nt)		identity%	
				Start	Stop	nt	aa
5’UTR	-	1	42	1	42	-	-
ORF 0	P0	1	798 (265)	43	840	80.10 (PuPV^**1**^)	67.17 (PuPV)
ORF 1	P1	1	1878 (625)	197	2074	87.33 (PABYV^**2**^-RSA1)	81.76 (PABYV-RSA)
ORF 1,2	P1, P2	1	3140 (1046)	197	3336	90.63 (PABYV-RSA)	87.76 (PABYV-RSA)
ORF 3a	p3a	1	138 (45)	3442	3579	97.10 (PABYV-RSA)	100 YP_010087205
ORF 3	P3	1	600 (199)	3560	4159	97.83 (PABYV-RSA)	96.98 (PuPV)
ORF 3,5	P3, p5	1	2076 (691)	3560	5635	90.14 (PuPV)	91.47 (PABYV-RSA)
ORF 4	P4	1	576 (191)	3588	4163	97.92 (PuPV)	95.29 (PuPV)
3’UTR		1	181	5636	5816	-	-

1; Pumpkin polerovirus 2; Pepo aphid-borne yellows virus

**Fig. 2:**
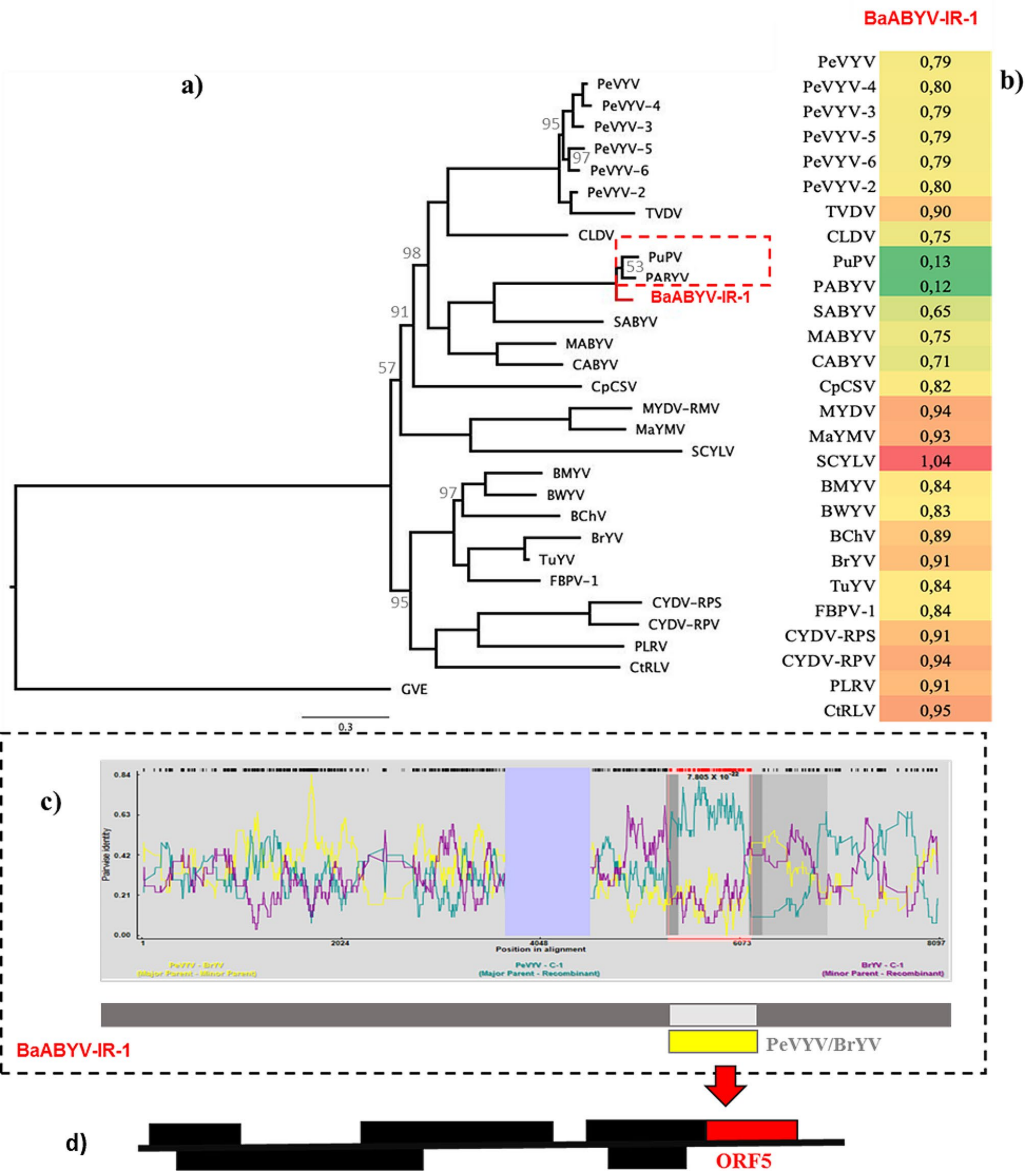
**(a)** The ML phylogenetic tree, rooted with Groundnut enation virus (GEV), is based on polerovirus sequences including BaABYV-IR-1 and the reference sequences of poleroviruses. The bootstrap values below 100 are indicated in the main nodes. **(b)** Heatmap displaying the pairwise distance between polerovirus reference nucleotide sequences and BaABYV-IR-1, calculated by MEGA X. The color intensity corresponds to distance levels, with dark red for highest and dark green for the lowest distance. **(c)** Recombination event in the BaABYV-IR-1 genome, identified through RDP analysis including reference polerovirus sequences and BaABYV-IR-1. This event was detected by RDP, MaxChi, Chimaera, SiScan, and 3Seq with a *P*-value less than 10^− 09^. The estimated major and minor parents were BrYV and PeVYV. **(d)** The recombination region is demarcated in red from positions 4,166 to 4,890 within the BaABYV-IR-1 sequence in ORF5

The reconstructed ML phylogenetic tree included 29 sequences, comprising the whole genome of the BaABYV-IR-1 isolate and reference sequences of other poleroviruses (see Appendix Table A2). The sequence matrix had 9,671 characters, 6,781 distinct patterns, and 5,405 parsimony-informative, 1,530 singleton, and 2,736 constant sites. The best-fit model determined by Bayesian Information Criterion (BIC) was GTR + F + I + G4. According to the phylogenetic tree, BaABYV-IR-1 strain is closely grouped with both PuPV and PABYV, suggesting that it may represent a divergent lineage within the genus *Polerovirus* (Fig. 2a). The phylogeny distinction is further supported by the calculated pairwise distances, which are represented in a heatmap (Fig. 2b), where BaABYV-IR-1’s proximity to PABYV compared to PuPV is notable. Based on this result, estimated that the major and minor parents were likely to be BrYV and PeVYV (Fig. 2c). This region of recombination was identified in the BaABYV-IR-1 isolate within ORF5 (Fig. 2d).

### Identification and phylogeny of SqVYV-IR-BA isolate

The findings indicated a high sequence similarity (98.42% identity) between contig C-2, designated *Squash vein yellowing virus* isolate IR-BA (SqVYV-IR-BA), and the reference genome of SqVYV, a member of the family *Potyviridae* (Table 2). Hence, following the ICTV guidelines, SqVYV-IR-BA is an isolate of the SqVYV species. To confirm these results, NextGenMap 0.5.0 was used to map trimmed reads of the SqVYV-IR-BA to the reference genome of SqVYV (NC_010521). Consequently, approximately 0.10% of the reads (42,715 nt) were successfully re-mapped to the reference genome. Table 4 provides a summary of the predicted ORF/region and amino acid length for each segment in the SqVYV-IR-BA isolate, including their start and stop nucleotide positions. Additionally, it features the identity percentages of nucleotides and amino acids, comparing sequences most similar to the SqVYV-IR-BA isolate with data sourced from GenBank.

**Table 4:** Genomic regions and ORF structure of SqVYV-IR-BA, and its similarity to some GenBank isolates

Predicted ORF/region	Length nt(aa)	Position(nt)		Identity%	
		Start	Stop	nt	aa
5’ UTR	117	1	117	94.69% ON229619.1	-
Polyprotein	9,525 (3174)	118	9642	98.45% ON229619.1	98.46% UQW19707.1
P1a	1,617 (539)	118	1734	94.64% ON229619.1	93.32% UQW19707.1
P1b	9,63 (321)	1735	2697	98.86% ON229619.1	99.38% UQW19707.1
P3	879 (293)	2698	3576	99.54% ON229619.1	99.32% UQW19707.1
6k1	156 (52)	3577	2732	100.00% ON013904.1	100.00% UQW19707.1
CI	1,887 (629)	3733	5619	99.26% ON229619.1	99.68% UQW19707.1
6k2	159 (53)	5620	5778	98.74% ON229619.1	100.00% UQW19707.1
VPg	576 (192)	5779	6354	99.48% ON229619.1	92.71% YP_001788998.1
NIa	699 (233)	6355	7053	99.43% ON229619.1	97.42% YP_001788999.1
NIb	1,512 (504)	7054	8565	99.07% ON229619.1	99.21% UQW19707.1
CP	1,074 (358)	8566	9639	99.35% ON229619.1	99.44% UQW19707.1
PIPO	237 (78)	3093	3329	100.00% ON229619.1	91.03% YP_006405420.1
3UTR	194	9643	9912 (9836)	99.48% ON229619.1	-

**Fig. 3:**
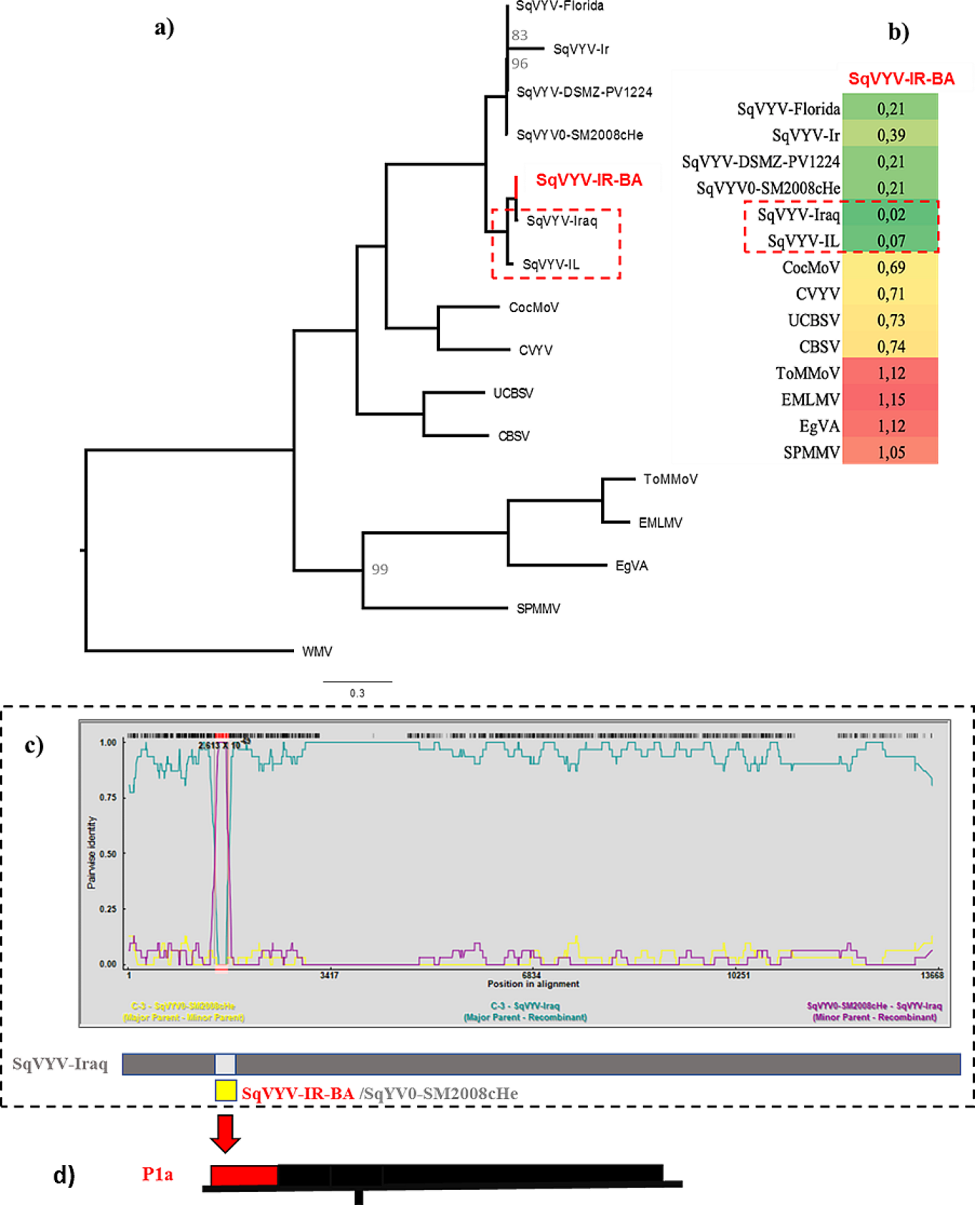
**(a)** The maximum likelihood phylogenetic tree based on reference sequences of imopoviruses and isolate SqVYV-IR-BA, rooted by WMV. The bootstrap values < 100 are indicated in the main nodes. **(b)** Heatmap based on a matrix including the pairwise distance between the nucleotide sequence of reference sequences of ipomoviruses with the sequence of SqVYV-IR-BA calculated by MEGA X. The color intensity indicates the level of distance between the species. Dark red indicates the highest number, while dark green represents the lowest number in the heatmaps. **(c)** Recombination event in genome of SqVYV-Iraq isolate detected by RDP analysis of a matrix including reference sequences of ipomovirus, available SqVYV isolates, and SqVYV-IR-BA. This event has been detected by RDP, GENECONV, BootScan, MaxChi, SiScan, and 3Seq with a P-value less than 10^− 09^. The estimated major and minor parents were SqVYV-IR-BA and SqYV0-SM2008cHe. **(d)** The schematic presentation of the SqVYV-IR-BA genome. The recombination region is shown in red color in positions 1,178 -1,371 in the SqVYV-IR-BA sequence located in the P1a region

The reconstructed ML tree was based on a matrix including 16 sequences (the SqVYV-IR-BA complete genome and the reference sequences of ipomoviruses; see Appendix, Table A3), with 13,668 characters, 7,892 distinct patterns, and 7,344, 2,823 and 3,501 parsimony-informative, singleton, and constant sites, respectively. The best-fit model based on BIC was GTR + F + I + G4. Figure 3b displays a heatmap created from a matrix that includes the pairwise distances between the nucleotide sequences of reference ipomoviruses and the SqVYV-IR-BA sequence.

The ML tree revealed two distinct clades of SqYVV isolates: one containing isolates from the USA and the other from the Middle East (Fig. 3a). Isolate SqVYV-IR-BA clustered with isolates from Middle East (SqVY-Iraq and SqVY-IL), signifying potential geographical influence on the genetic variability (Fig. 3c). The recombination detection test did not identify SqVYV-IR-BA as a recombinant isolate (Fig. 3d).

### Identification and phylogeny analysis of WMV-IR-BA

Bioinformatic analysis identified a contig labeled C-3, designated as WMV-IR-BA, with a length of 3,757 nucleotides. This contig exhibited approximately 96% identity with WMV isolates CHI87-620 and VE10-099 which are documented in GenBank under accession numbers EU660580 and KC292915 respectively.

The contig known as WMV-IR-BA was found to contain an incomplete ORF encompassing four genomic regions: NIa-VPg (nuclear inclusion VPg protein), NIa-Pro (nuclear inclusion protein), NIb (nuclear inclusion b) and CP (coat protein gene). The contig was mapped to the reference genome of WMV (NC_006262) using NextGenMap 0.5.0, confirming the initial findings. Table 5 outlines the predicted ORF/region, amino acid length, and the positions of each segment in the WMV-IR-BA isolate. It also presents the identity percentages for nucleotides and amino acids, comparing sequences closely related to the WMV-IR-BA isolate as found in GenBank.

**Table 5:** ORF structure of the WMV-IR-BA isolate, and its similarity to some GenBank isolates

Predicted ORF/region	Length nt(aa)	Position(nt)		Identity%	
		Start	Stop	nt	aa
Polyprotein	3566 (1137)	1	3566	96.38% EU660580	98.42% UCC70027
NIa-VPg	440 (146)	1	440	95.45% EU660580	94.52% YP_077274
VPg-pro	729 (243)	441	1169	96.57% KC292915	97.94% YP_077275
NIb	1551 (517)	1170	2720	97.80% D13913.1	98.84% UCC70027
CP	843 (283)	2721	3563	96.56% AJ579481	97.86% AAX89506

**Fig. 4:**
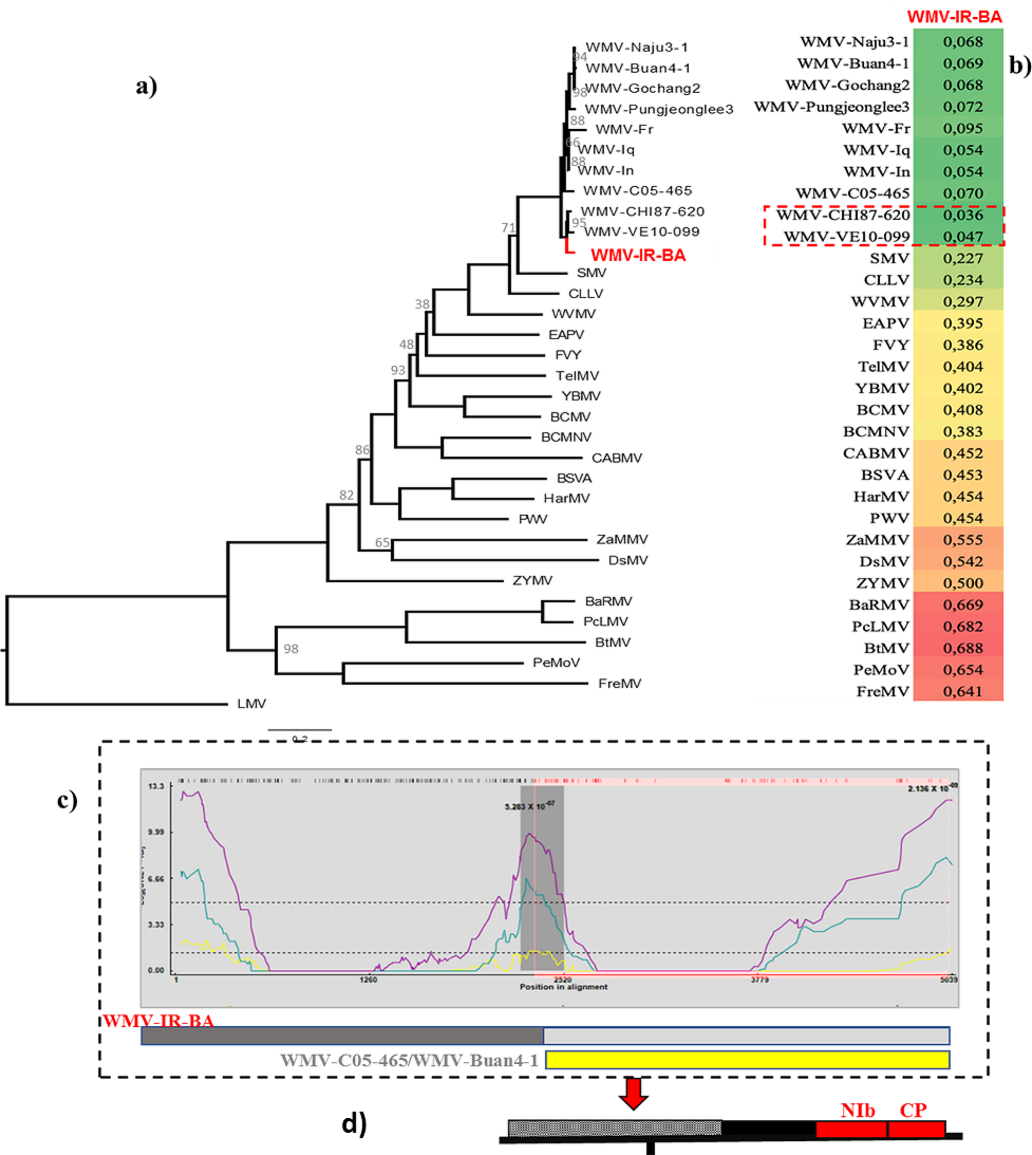
**(a)** The maximum likelihood phylogenetic tree based on partial sequence of the *Watermelon mosaic virus* isolate (WMV-IR-BA) using 10 WMV isolates, reference sequence of potyviruses and subgroup BCMV, rooted by LMV. The bootstrap values < 100 are indicated in the main nodes. **(b)** Heatmap based on a matrix including the pairwise distance between the nucleotide sequence of reference sequences of potyviruses, subgroup BCMV with the sequence of the WMV-IR-BA isolate calculated by MEGA X. The color intensity indicates the level of distance between the species. Dark red indicates the greatest divergence, while dark green indicates the least. **(c)** Recombination event in genome of the WMV-IR-BA isolate detected by RDP analysis of a matrix including reference sequences of potyviruses, 10 WMV isolates, and the WMV-IR-BA isolate. This event has been detected by GENECONV, BootScan, MaxChi, SiScan, and 3Seq with a *P*-value less than 10^− 06^. The estimated major and minor parents were WMV-C05-465 and Buan 4 − 1. **(d)** The schematic presentation of the WMV-IR-BA genome. The recombination region is in red color in positions 2,241-3,757 in the WMV-IR-BA sequence, located in the NIb and CP regions

The reconstructed ML-tree included nucleotide sequences from the complete genome of WMV-IR-BA, 10 closely related WMV isolates, along with reference sequences from the Bean common mosaic virus (BCMV) subgroup of potyviruses (Appendix, Table A4). This selection was made due to the close relationship between WMV and other members of the BCMV subgroup [25]. The phylogenetic tree was based on a matrix including 33 sequences with 5,039 characters, 3,001 distinct patterns, 2,573, 510, and 1,956 parsimony-informative, singleton, and constant sites, respectively. The best-fit model for this dataset, based on BIC was GTR + F + I + G4. Within this framework, WMV-IR-BA and two isolates from South America formed a well-supported clade (Fig. 4a). The heatmap based on the distance matrix between WMV-IR-BA and other reference sequences of subgroup BCMV has been displayed in Fig. 4b. The WMV-IR-BA isolate was identified as a recombinant through RDP analysis, with major and minor parents traced back to France and South Korea (Fig. 4c). The recombination region was identified in the WMV-IR-BA isolate within the NIb and CP regions (Fig. 4d).

### Identification and phylogeny of the CiaRV-IR-BA isolate

The contig labeled C-4, referred to as citrus-associated rhabdovirus isolate IR-BA (CiaRV-IR-BA), has a length of 13,443 nucleotides. It has a maximum identity of 80.82% with the CiaRV (MT302545), classified in the genus *Cytorhabdovirus* within the family *Rhabdoviridae* [26]. The nucleotide and amino acid identity between CiaRV-IR-BA and the closest strain ranged between 80 and 90% and 85–95% respectively for nearly all ORFs. Notably, ORF4 exhibits a lower identity, with 73% at the nucleotide level and 65% (Table 6). For further analyses, the trimmed reads were mapped to the reference genome of CiaRV (MT302542) using NextGenMap 0.5.0. According to the results, 14,580 reads were re-mapped on the reference genome.

**Table 6:** Genomic regions and ORF structure of the CiaRV-IR-BA (the coting-4), and its similarity to selected GenBank isolates

Predicted ORF/region	ORF product		Positions(nt)		Identity%	
		Length nt(aa)	Start	Stop	nt	aa
3’UTR		136	1	136	80.88% MT302542	-
ORF1	N	1347 (448)	137	1483	82.82% OP689651.1	89.58% QMS92539
ORF2	P	1326 (441)	1675	3000	81.53% MT302544	85.52% UYP40104
ORF3	P3	570 (189)	3163	3732	81.05% MK202584	93.12% QMS92551
ORF5	M	645 (214)	4278	4922	81.83% MT302546	93.93% QXF30349
ORF6	G	1560 (519)	5141	6700	80.07% MT302546	89.21% UYP40108
ORF7	L	6342 (2113)	6953	13,294	81.05% MT302545	93.23% UOF75634
ORF4	P4	240 (79)	3749	3988	72.53% MK202584	64.56% UYP40106.
5’UTR		149	13,295	13,443	91.49%OP689651	

The reconstructed ML tree was based on a matrix including 42 sequences, which encompassed the whole genome of CiaRV-IR-BA and the reference sequences of cytorhabdoviruses, along with 10 CiaRV isolates closely related to CiaRV-IR-BA (see Appendix, Table A5). This matrix contained 25,146 characters, 20,731 distinct patterns, and 17,115, 3,585, and 4,446 parsimony-informative, singleton, and constant sites, respectively. The optimal model for this dataset based on BIC, was GTR + F + I + G4.

**Fig. 5:**
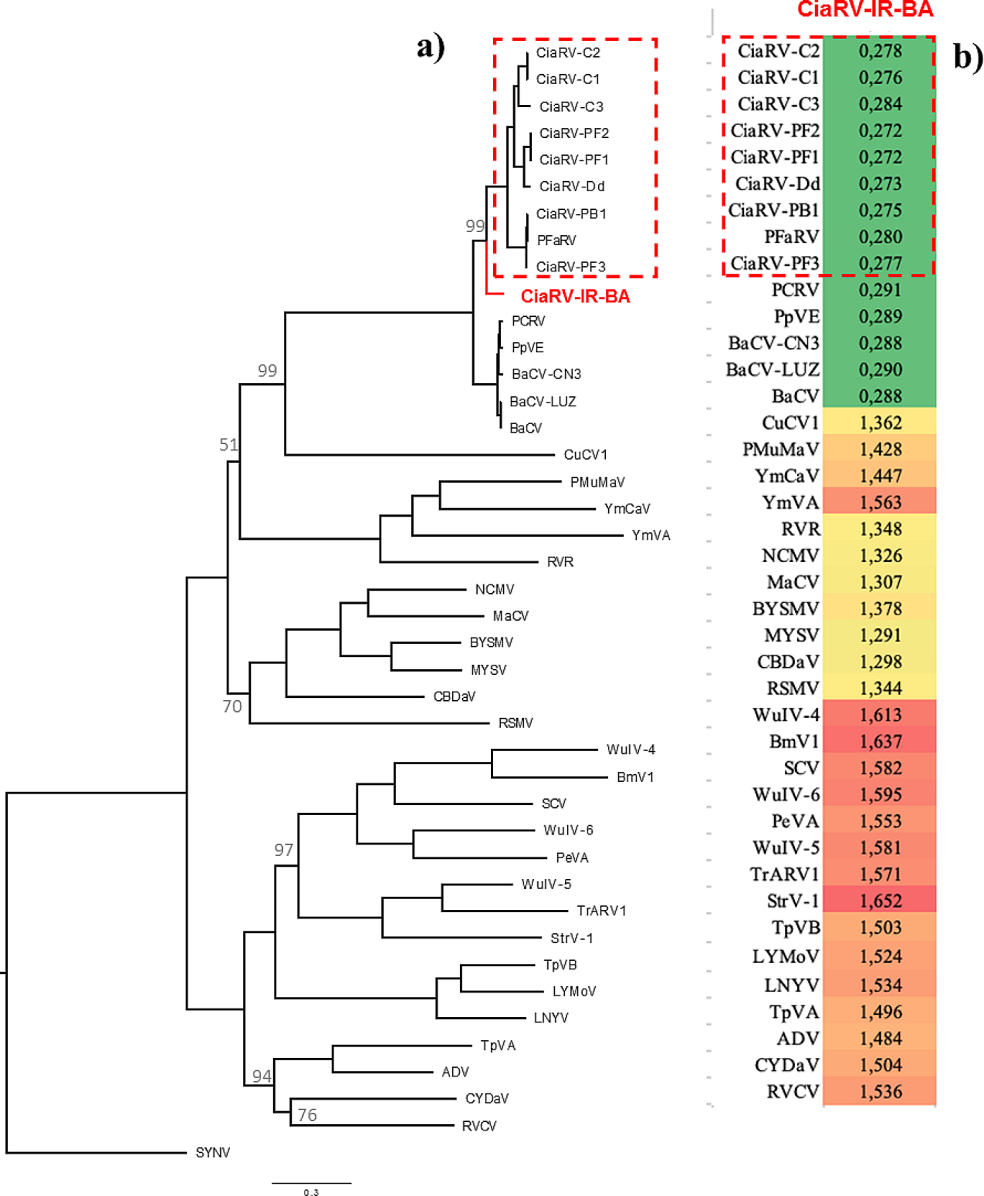
**(a)** The maximum likelihood phylogenetic tree based on the partial sequence of the isolate CiaRV-IR-BA, 10 CiaRV isolates, and the reference sequences of cytorhabdoviruses rooted by SYNV. The bootstrap values < 100 are indicated in the main nodes. **(b)** Heatmap based on a matrix including the pairwise distance between the nucleotide sequence of the reference sequences of cytorhabdoviruses with the sequence of CiaRV-IR-BA calculated by MEGA X. The color intensity indicates the level of the distance between the species. Dark red indicates the highest number, while dark green represents the lowest number in the heatmap

The phylogenetic tree highlighted that CiaRV isolates, including the CiaRV-IR-BA, diverged significantly from other sequences, forming a unique clade, as shown in Fig. 5a. The heatmap based on the distance matrix between CiaRV-IR-BA and other reference sequences of the subgroup *Cytorhabdovirus* has been displayed in Fig. 5b. The CiaRV-IR-BA isolate is not a recombinant nor a parent of recombinants based on RDP analysis (Fig. 5b).

### RT-PCR assay for verification of RNA-Seq data

In our RNASeq analysis, we primarily identified four large contigs corresponding to the four different viruses: BaABYV-IR, SqVYV-IR-BA, WMV-IR-BA, and CiaRV-IR-BA (Table 2). To confirm the results of RNA-Seq analysis, we conducted RT-PCR assays using virus-specific primers on the same pooled sample that previously prepared for NGS analysis, comprising seven bitter apple plants. As listed in Table 1, these primers were specifically designed to target distinct regions of each viral genome, thereby enabling their precise and unambiguous identification.

The RT-PCR successfully yielded amplified products of the expected sizes for each virus. Subsequent sequencing of these amplified products validated the presence of BaABYV-IR, SqVYV-IR-BA, WMV-IR-BA, and CiaRV-IR-BA in the bitter apple sample (sequencing data not provided).

## Discussion

Metagenomics has emerged as a powerful tool for detecting viruses in plants, even when no symptoms are visible. It allows the simultaneous analysis of all genetic material in a sample, detecting both known and unknown viruses, as well as multiple viruses in a single sample [13]. Despite these advantages, the technique is not without limitations. One significant challenge is the lack of virion-enriched methods, which can hinder the acquisition of high-quality, representative viral samples, thus introducing bias into the data [12]. Moreover, the genetic diversity of viral communities often complicates data interpretation, especially for uncharacterized viruses not in databases. The computational tools for metagenomic analysis are continually evolving but require significant computational power [27].

To decrease these biases in our study, we implemented pool sampling by combining samples from available symptomatic plants, aiming to enhance virion enrichment. We also integrated a purification step to improve the quality of the genetic material for subsequent NGS analysis. Utilizing the Illumina HiSeq 6000 sequencing platform, known for its high accuracy and quality, we sought to improve detection accuracy. Furthermore, to ensure the reliability of our analysis, we employed three different *de novo* assembly tools and a mapping method to validate the generated contig.

In our study, we applied metagenomic analysis to examine viral presence in *C. colocynthis*, a wild plant species extensively cultivated in southern Iran. Building upon a previous research which identified WMV from bitter apple plants in the Jiroft region, our study aimed to explore a wider range of potential viruses in bitter apple plants showing viral symptoms in the Jiroft region. In addition to WMV, other studies in Iran have detected the presence of Papaya ring spot virus-type W and cucurbit aphid-borne yellows *virus* in this plant [4–5]. These findings indicate that this plant species can act as a viral reservoir affecting agricultural crops [4–8].

In current research, through *de novo* assembly, we identified several contigs with high similarity to known viruses in the genera *Polerovirus*, *Impomovirus*, *Potyvirus*, and *Cytorhabdovirus*. The species demarcation, as outlined by the ICTV, facilitated the classification of detected isolates. Notably, we identified a novel polerovirus species, BaABYV-IR-1, and documented the first occurrence of the Papaya cytorhabdovirus in Iran. These findings add to global inventory of recently identified Polerovirus and Cytorhabdovirus species [28–33]. The advancements in molecular biology and sequencing technologies has facilitated these discoveries [13]. The BaABYV, belonging to the genus *Polerovirus* classified within the family *Solemoviriade*, infects a variety of plant species including dicots and monocots. Its genome comprises a linear, single-stranded RNA containing ORFs 0, 1, 2, 3a, 3, 4 and 5 [34–35]. Given that the translated ORFs 0, 1, and 2 of BaABYV-IR-1 exhibit amino acid sequence similarities ranging from 68 to 88% with publicly available viral species in the family *Luteovoridae*. By meeting the ICTV threshold, which necessitates over 10% divergence in amino acid sequences of any gene product for special delineation [36], BaABYV-IR-1 has been classified as a new virus species. This isolate has been deposited in the GenBank database under the accession number OR266512. Further investigations into the metagenomics data revealed the presence of three additional virus species.

The BLAST analysis of contig C2 demonstrates a remarkably high level of similarity, as per the taxonomic criteria defined by ICTV. Such findings conclusively categorize it within the genus *Ipomovirus*, pinpointing it as the SqVYV. The only Iranian SqVYV isolate previously recorded in the GenBank database (SqVYV-Ir, accession number KU953950) was notably clustered in the clade predominantly containing USA isolates. This presents an intriguing divergence compared to SqVYV-IR-BA and other isolates originating from the Middle East. Lacking additional published data on SqVYV-Ir precludes further analysis. SqVYV is phylogenetically linked as a sister group to both the Coccinia mottle virus (CocMoV) and Cucumber vein yellowing virus (CVYV). These affiliations are not merely taxonomical but also show up as similar symptoms in the host plants. All three viruses belong to the same genus and share a restricted host range, limited explicitly to plants in the *Cucurbitaceae* family [37]. This isolate was submitted to GenBank with the accession number OR232212.

Analysis of contig C3 revealed its relationship with the Potyvirus subgroup, elucidating its identification as a WMV isolate. Following the ICTV guidelines, the WMV-IR-BA isolate, with an identity exceeding 90% with existing WMV isolates, qualifies as a member of the WMV species within the family *Potyviridae* [38–39] This isolate has also been submitted to GenBank under the accession number OR345349. The current study has undertaken the first examination of both Iranian isolates, SqVYV-IR-BA and WMV-IR-BA, regarding their phylogenetics and recombination attributes.

Cytorhabdoviruses are enveloped viruses with single-stranded, negative RNA genomes that infect a range of hosts, including plants, animals, and insects [40–42]. The genome typically spans approximately 12.2 to 14.5 kb and encodes a variety of proteins, including the structural proteins (nucleocapsid and envelope), enzymes (RNA polymerase and ribonucleoprotein), and accessory proteins (such as movement proteins or virulence factors) [40, 43–44]. These viruses encapsulate their RNA within the nucleocapsid protein, which is then surrounded by a host-derived lipid membrane to complete virus particle formation [43, 45]. The analysis of contig C-4, designated as CiaRV-IR-BA, revealed it contains five major ORFs typical of rhabdoviruses, coding for nucleoprotein (N), phosphoprotein (P), a putative movement protein (P3), hypothetical protein (P4), matrix (M), glycoprotein (G), and an RNA-dependent RNA polymerase (L) [44]. Species demarcation within the genus *Cytorhabdovirus* is based on genome sequence identity below 75% and amino acid sequence identity under 80% across all cognate ORFs [43]. In the case of CiaRV-IR-BA, nearly all ORFs are more than 85.5% similar to existing CiaRV sequences. This suggests that CiaRV-IR-BA can be considered an isolate of the species *Papaya cytorhabdovirus*, as proposed by Zhang et al. [46]. However, the lower identity scores for ORF4 at 73 and 65% are notable, possibly pointing to a unique or fast-evolving protein [47–48]. This variability could contribute to differential host specificity, virulence, or other ecologically significant traits [49]. The isolate has been submitted to GenBank with the accession number OR232213.

This research has elucidated the recombination dynamics within the genomes of BaABYV-IR-1 and WMV-IR-BA, emphasizing their potential implications for viral evolution and host adaptation. Recombination detection analysis revealed evidence of recombination in ORF5 of BaABYV-IR-1 genome, a gene that encodes for a movement protein crucial for viral replication and host adaption [50]. This suggests that BaABYV-IR-1 could potentially expand its host and vector range, a phenomenon observed in other poleroviruses, like the *Soybean chlorotic leafroll virus* (SbCLRV) [51]. The recombinant region spans positions 4,166 to 4,890 within the BaABYV-IR-1 genome, indicating a genetic exchange between Brassica yellows virus (BrYV) and Pepper vein yellows virus (PeVYV) as the major and minor parent, respectively. For WMV-IR-BA, the pinpointed recombination event within cistron NIb-CP, validates earlier studies labeling this region as a recombination hotspot [52]. This could have significant implications for the ability of the virus to adapt to new hosts and environments. Our study also examined recombination in SqVYV isolates. Remarkably, while SqVYV-IR-BA did not display recombination itself, it acted as a major parent in a recombination event in the P1b region for an isolate from Iraq, challenging the previously observed trend of high recombination rates in American isolates within P1a region [53]. The observed lower genetic diversity within SqVYV is an irregularity among ipomoviruses [53], potentially is attributable to negative selection pressures or a genetic bottleneck event similar to the evolutionary pattern of cucumber yellow vein virus in Spain [54]. In contrast to these findings, the CiaRV-IR-BA isolate did not exhibit any recombination nor it serve as a parent in any recombinant forms, hinting at a possibly stable evolutionary path that may be due to limited genetic diversity or host specificity. Our study not only sheds light on the recombination patterns of various viruses but also raises important questions about the evolutionary mechanisms that drive these events. The absence of recombination in CiaRV-IR-BA, for instance, could be indicative of a stable evolutionary lineage or could suggest that the virus has not yet been exposed to conditions that facilitate recombination [55–57]. These findings underscore the need for further research to understand the ecological and evolutionary dynamics that influence viral recombination, host adaptation, and the emergence of new viral strains.

### Future research and implications

Our study provides robust validation of specific viruses in the bitter plant samples, thanks to the use of advanced techniques and cross-validation methods. This underscore the reliability of high-throughput sequencing methods like RNA-Seq in virological studies and paves the way for targeted interventions and a deeper understanding of virus-plant interactions. Given the strong amino acid sequence similarity and robust RNA-Seq data validation via RT-PCR, further research is essential. While, our study confirmed the presence of specific viruses using advanced techniques, it also highlighted several areas for future investigations. The limited geographic scope and sample size (*n* = 7) point to the need for more extensive research to achieve broader and more generalizable conclusions. Future studies should consider a larger sample size and geographic range, particularly beyond the Jiroft region.

A notable finding was the common viral symptoms such as dwarfing, leaf crinkling, and mild chlorosis observed in our samples, further complicated by mixed infections. This underscores the need for further research focused on isolating individual viruses and conducting biological assays to understand the role of each virus and its interaction with bitter apple plants. Additionally, the potential impact of these viruses on the medicinal properties of bitter apple remains an unexplored area of research.

The observed proximity of bitter apple to citrus trees in the Jiroft region, raises questions about the transmission of Citrus-associated rhabdovirus (CiaRV) to bitter apple plants, possibly through common vectors. While various insects like aphids, planthoppers, and leafhoppers are known to transmit cytorhabdoviruses [58], and whiteflies have been identified as vectors for Bean-associated cytorhabdovirus [59], the specific vector responsible for CiaRV transmission remains unidentified. Our observations suggest a potential transmission risk, but more research is needed to confirm this and to understand the virus transmission dynamics in this agroecosystem.

Additionally, our study is temporal scope offers opportunities for future research. We plan to investigate the viral community in bitter apple plants across different seasons and time periods, considering their potential as annual or perennial. This will be crucial for understanding seasonal changes in viral prevalence, which is vital for developing effective disease management strategies. The influence of local climate, marked by mild winters and hot, humid summers, on virus prevalence should also be considered in these studies.

### Electronic supplementary material

Below is the link to the electronic supplementary material.

**Supplementary Material 1**: Metatranscriptome analysis of symptomatic bitter apple plants revealed mixed viral infections with a putative novel polerovirus

## Data Availability

Sequencing data generated in this study have been deposited in the NCBI Sequence Read Archive database under accessions PRJNA1005066.

## Abbreviations

NGS: Next generation Sequencing
ICTV: International Committee on Taxonomy of Viruses
MSAs: Multiple sequence alignments
MLtree: Maximum likelihood phylogenetic tree
RT: Reverse Transcription
NCBI: National Center for Biotechnology Information
RVDB: Reference Viral DataBase
SqVYV: Squash vein yellowing virus
CiaRV: Citrus-associated rhabdovirus
BaABYV: Bitter apple aphid-borne yellows virus
WMV: Watermelon mosaic virus
CABYV: Cucurbit aphid-borne yellows virus
SqMV: Squash mosaic virus
PRSV-W: Papaya ring spot virus-type W
CMV: Cucumber mosaic virus
CocMoV: Coccinia mottle virus
CVYV: Cucumber vein yellowing virus
ZYMV: Zucchini yellow mosaic virus
PABYV: Pepo aphid-borne yellows virus
PuPV: Pumpkin polerovirus
GEV: Groundnut enation virus
BrYV: Brassica yellows virus
PeVYV: Pea enation mosaic virus
LMV: Lettuce mosaic virus
BCMV: Bean common mosaic virus
