# Supplementary material for: Metatranscriptome analysis of symptomatic bitter apple plants revealed mixed viral infections with a putative novel polerovirus
Source: BMC Genomics. 2024 Feb 15;25:181. doi: 10.1186/s12864-024-10057-z (PMC10868029; doi:10.1186/s12864-024-10057-z)
Supplement: Supplementary file 1 — Supplementary Material 1: Metatranscriptome analysis of symptomatic bitter apple plants revealed mixed viral infections with a putative novel polerovirus [file 12864_2024_10057_MOESM1_ESM.docx]

**Appendix: Metatranscriptome analysis of symptomatic bitter apple plants revealed mixed viral infections with a putative novel polerovirus**

Table A1. Comparative analysis of *de novo* assembly results using three different assemblers

| **Tool** | **contigs** | **Sum length** | **N50** | **Minimum length** | **Maximum length** | **Average length** |
| --- | --- | --- | --- | --- | --- | --- |
| Trinity | 77,188 | 81,323,445 | 1,678 | 201 | 14,869 | 1,054 |
| SPAdes | 36,784 | 471,07,524 | 1,834 | 86 | 27,722 | 1,281 |
| CLC | 51,435 | 40,090,519 | 1,200 | 200 | 18,269 | 779 |

Table A2. Reference sequences of available poleroviruses used in this study

| **GenBank accession number** | ***Species*** | **Isolate** | **Abbreviation** | **Length of genome (nt)** |
| --- | --- | --- | --- | --- |
| NC_055513 | *Pumpkin polerovirus* | PuPV | PuPV | 5810 |
| NC_002766 | *Beet chlorosis virus* |  | BChV | 5776 |
| NC_003491 | *Beet mild yellowing virus* |  | BMYV | 5722 |
| NC_004756 | *Beet western yellows virus* |  | BWYV | 5666 |
| NC_016038 | *Brassica yellows virus* | BrYV-ABJ | BrYV | 5666 |
| NC_006265 | *Carrot red leaf virus* |  | CtRLV | 5723 |
| NC_002198 | *Cereal yellow dwarf virus-RPS* |  | CYDV-RPS | 5662 |
| NC_004751 | *Cereal yellow dwarf virus-RPV* |  | CYDV-RPV | 5723 |
| NC_014545 | *Cotton leafroll dwarf virus* |  | CLDV | 5866 |
| NC_010809 | *Melon aphid-borne yellows virus* |  | MABYV | 5674 |
| NC_030225 | *Pepo aphid-borne yellows virus* | RSA BB Marrow | PABYV | 5813 |
| NC_015050 | *Pepper vein yellows virus* |  | PeVYV | 6244 |
| [HM439608](https://www.ncbi.nlm.nih.gov/nuccore/HM439608) | *Pepper vein yellows virus 2* | IS | PeVYV-2 | 6244 |
| [KP326573](https://www.ncbi.nlm.nih.gov/nuccore/KP326573) | *Pepper vein yellows virus 3* | HN | PeVYV-3 | 6244 |
| [KU999109](https://www.ncbi.nlm.nih.gov/nuccore/KU999109) | *Pepper vein yellows virus 4* | 12KNX1 | PeVYV-4 | 6244 |
| [KY523072](https://www.ncbi.nlm.nih.gov/nuccore/KY523072) | *Pepper vein yellows virus 5* | Spain-Almeria 2-2013 | PeVYV-5 | 6125 |
| [LT559483](https://www.ncbi.nlm.nih.gov/nuccore/LT559483) | *Pepper vein yellows virus 6* |  | PeVYV-6 | 6096 |
| NC_001747 | *Potato leafroll virus* |  | PLRV | 5987 |
| NC_018571 | *Suakwa aphid-borne yellows virus* | SABYV-TW19 | SABYV | 5843 |
| NC_000874 | *Sugarcane yellow leaf virus* |  | SCYLV | 5899 |
| NC_003743 | *Turnip yellows virus* |  | TuYV | 5641 |
| NC_055495.1 | *Faba bean polerovirus 1* | 5253 | FBPV-1 | 5631 |
| NC_021484.1 | *Maize yellow dwarf virus-RMV* |  | MYDV | 5612 |
| NC_003688.1 | *Cucurbit aphid-borne yellows virus* |  | CABYV | 5669 |
| NC_010732.1 | *Tobacco vein distorting virus* |  | TVDV | 5920 |
| NC_008249.1 | *Chickpea chlorotic stunt virus* |  | CpCSV | 5900 |
| MT809617.1 | *Maize yellow mosaic virus* | MaYMV If15-TZA | MaYMV | 5641 |
| NC_011106 | *Grapevine virus E* |  | GVE | 7564 |

Table A3. Reference sequences of available ipomoviruses used in this study

| GsnBank accession number | Species | Isolate | Abbreviation | Length of genome (nt) |
| --- | --- | --- | --- | --- |
| NC_010521 | *Squash vein yellowing virus* | Florida | SqVYV-Florida | 9836 |
| KT721735 | *Squash vein yellowing virus* | IL | SqVYV-IL | 9831 |
| KU953950 | *Squash vein yellowing virus* | SqVYV-Ir | SqVYV-Ir | 9834 |
| JF897996 | *Squash vein yellowing virus* | SM2008cHe | SqVYV-SM2008cHe | 9836 |
| ON229619 | *Squash vein yellowing virus* | SVYV/Iraq | SqVYV-Iraq | 9832 |
| ON013904 | *Squash vein yellowing virus* | DSMZ PV-1224 | SqVYV-DSMZ-PV1224 | 9834 |
| NC_006262 | *Watermelon mosaic virus* | - | WMV | 10035 |
| NC_038920 | *Tomato mild mottle virus* | - | TOMMOV | 9283 |
| NC_030840 | *Coccinia mottle virus* | Su12-25 | CocMoV | 9778 |
| NC_012698 | *Cassava brown streak virus* | KOR6 | CBSV | 8995 |
| NC_014791 | *Ugandan cassava brown streak virus* | UG | UCBSV | 9070 |
| NC_006941 | *Cucumber vein yellowing virus* | ALM32 | CVYV | 9751 |
| NC_003797 | *Sweet potato mild mottle virus* | - | SPMMV | 10818 |
| MW503940 | *Eggplant mild leaf mottle virus* | - | EMLMV | 9280 |
| LC552941 | *Eggplant virus A 2_70* | EgVA | EgVA | 9328 |

Table A4. Reference sequences of available potyviruses used in this study

| **GenBank accession number** | ***Species*** | **Isolate** | **Abbreviation** | **Length of genome (nt)** |
| --- | --- | --- | --- | --- |
| NC_002634 | *Soybean mosaic virus* | N | SMV | 9588 |
| EF105299 | *Calla lily latent virus* | E49 | CLLV | 9728 |
| NC_007216 | *Wisteria vein mosaic virus* | Beijing | WVMV | 9695 |
| NC_010954 | *fritillary virus Y* | Pan'an | FVY | 9656 |
| NC_007728 | *East Asian Passiflora virus* | AO | EAPV | 10046 |
| NC_009742 | *Telosma mosaic virus* | Hanoi | TelMV | 9689 |
| NC_004047 | *Bean common mosaic necrosis virus* | Michigan | BCMNV | 9612 |
| NC_016441 | *Yambean mosaic virus* | SR | YBMV | 9649 |
| NC_003397 | *Bean common mosaic virus* | R | BCMV | 9992 |
| NC_004013 | *Cowpea aphid-borne mosaic virus* | CABMV-Z | CABMV | 9465 |
| NC_019415 | *Blue squill virus A* | SW3 | BSVA | 9842 |
| NC_015394 | *Hardenbergia mosaic virus* | HarMV-57.2 | HarMV | 9682 |
| NC_014790 | *Passion fruit woodiness virus* | PWV-MU2 | PWV | 9682 |
| NC_003224 | *Zucchini yellow mosaic virus* | TW-TN3 | ZYMV | 9591 |
| NC_011560 | *Zantedeschia mild mosaic virus* |  | ZaMMV | 9973 |
| NC_003537 | *Dasheen mosaic virus* | M13 | DsMV | 10038 |
| NC_009741 | *Basella rugose mosaic virus* | AC | BaRMV | 9804 |
| DQ851494 | *Peace lily mosaic virus* | Haiphong | PcLMV | 9882 |
| NC_005304 | *Beet mosaic virus* | BtMV-Wa | BtMV | 9591 |
| NC_002600 | *Peanut mottle virus* |  | PeMoV | 9709 |
| NC_014064 | *Freesia mosaic virus* | Freesia sp. | FreMV | 9489 |
| NC_006262 | *Watermelon mosaic virus* | WMV-Fr | WMV-Fr | 10035 |
| EU660580 | *Watermelon mosaic virus* | CHI87-620 | WMV-CHI87-620 | 10045 |
| KC292915 | *Watermelon mosaic virus* | VE10-099 | WMV-VE10-099 | 10039 |
| MT780536 | *Watermelon mosaic virus* | WMV-Iq | WMV-Iq | 10043 |
| MT780537 | *Watermelon mosaic virus* | WMV-In | WMV-In | 9999 |
| KU240100 | *Watermelon mosaic virus* | Naju3-1_2012 | WMV-Naju3-1 | 9900 |
| KU240096 | *Watermelon mosaic virus* | Gochang2_2012 | WMV-Gochang2 | 9913 |
| KT992070 | *Watermelon mosaic virus* | Buan4-1_2013 | WMV-Buan4-1 | 10111 |
| JF273460 | *Watermelon mosaic virus* | C05-465 | WMV-C05-465 | 10046 |
| KU240102 | *Watermelon mosaic virus* | Pungjeonglee3_2012 | WMV-Pungjeonglee3 | 9893 |
| NC_003605 | *Lettuce mosaic virus* |  | LMV | 10080 |

Table A5. Reference sequences of available cytorhabdoviruses used in this study

| **GenBank accession number** | **Species** | **Isolate** | **Abbreviation** | **Length of genome (nt)** |
| --- | --- | --- | --- | --- |
| C-5 |  |  | C-5 | 13443 |
| MH982250 | *Trifolium pratense virus A* | 29/15/1 | TpVA | 12430 |
| NC_031225 | *Wuhan insect virus 4* | YCYC03 | WuIV-4 | 13490 |
| BK014479 | *Bacopa monnieri virus 1* | India | BmV1 | 13332 |
| MH982249 | *Trifolium pratense virus B* | 1/2014 | TpVB | 13875 |
| NC_031227 | *Wuhan insect virus 5* | YCYC02 | WuIV-5 | 12734 |
| MN872813 | *Paper mulberry mosaic associated virus* | SWU | PMuMaV | 13736 |
| MW239071 | *Papaya virus E* | Macroptilium | PpVE | 13481 |
| MW039593 | *Chrysanthemum yellow dwarf associated virus* | cq | CYDaV | 14086 |
| NC_034551 | *Colocasia bobone disease-associated virus* | SI | CBDaV | 12193 |
| MT381995 | *Cucurbit cytorhabdovirus 1* | ND4 | CuCV1 | 13069 |
| NC_055505 | *Yerba mate chlorosis-associated virus* | Montecarlo | YmCaV | 12595 |
| MN317172 | *Strawberry virus 1* | 1/2017 | StrV-1 | 13989 |
| MZ962213 | *Strawberry crinkle virus* | Heidelberg (Hb-A1) | SCV | 14547 |
| NC_031232 | *Wuhan insect virus 6* | SXCC01-1 | WuIV-6 | 14191 |
| NC_002251 | *Northern cereal mosaic virus* | - | NCMV | 13222 |
| NC_028244 | *Barley yellow striate mosaic virus* | Hebei | BYSMV | 12706 |
| NC_011532 | *Lettuce yellow mottle virus* | - | LYMoV | 12926 |
| NC_007642 | *Lettuce necrotic yellows virus* | - | LNYV | 12807 |
| KY965147 | *Maize-associated cytorhabdovirus* | Peru | MaCV | 11877 |
| NC_055454 | *Maize yellow striate virus* | - | MYSV | 12654 |
| NC_028237 | *Alfalfa dwarf virus* | Manfredi | ADV | 14494 |
| NC_040786 | *Rice stripe mosaic virus* | GD-LD | RSMV | 12774 |
| NC_018381 | *Persimmon virus A* | persimmon isolate | PeVA | 13467 |
| MT952336 | *Rose virus R* | MDR92016 | RVR | 13601 |
| MK240091 | *Raspberry vein chlorosis virus* | Hutton_1 | RVCV | 14512 |
| BK011194 | *Trichosanthes associated rhabdovirus 1* | Shenzhen | TrARV1 | 12783 |
| MN781667 | *Yerba mate virus A* | Gob. Virasoro | YmVA | 14961 |
| MT302547 | *Citrus-associated rhabdovirus* | C3 | CiaRV-C3 | 13526 |
| MT302546 | *Citrus-associated rhabdovirus* | C2 | CiaRV-C2 | 13498 |
| MT302545 | *Citrus-associated rhabdovirus* | C1 | CiaRV-C1 | 13496 |
| MT302543 | *Citrus-associated rhabdovirus* | PF3 | CiaRV-PF3 | 13505 |
| MT302542 | *Citrus-associated rhabdovirus* | PF2 | CiaRV-PF2 | 13485 |
| MT302541 | *Citrus-associated rhabdovirus* | PF1 | CiaRV-PF1 | 13485 |
| MT302544 | *Citrus-associated rhabdovirus* | PB1 | CiaRV-PB1 | 13497 |
| OP689651 | *Citrus-associated rhabdovirus* | Dd_DN69067 | CiaRV-Dd | 13463 |
| MZ272468 | *Passionfruit-associated rhabdovirus* | YN | PFaRV | 13546 |
| MK202584 | *Bean-associated cytorhabdoviru* | BaCV-BR-GO | BaCV | 13467 |
| MT792847 | *Bean-associated cytorhabdovirus* | CN3 | BaCV-CN3 | 13427 |
| MT811775 | *Cytorhabdovirus caricae* | BaCV-LUZ | BaCV-LUZ | 13467 |
| NC_055504 | *Papaya cytorhabdovirus* | Los Rios_Ec | PCRV | 13469 |
| NC_001615 | *Sonchus yellow net virus* | - | SYNV | 13720 |
